# Influence of Silver Nanoparticle Incorporation on the Flexural Strength and Color Stability of Heat-Polymerized Polymethyl Methacrylate (PMMA) Denture Base Resin: An In Vitro Study

**DOI:** 10.7759/cureus.114781

**Published:** 2026-08-19

**Authors:** Bhavana T.V, Raja Reddy N, Sumedha Sahanasree Dasari, Rashmitha Regoti, Ramisetty Saranya, Rajitha Reddy K

**Affiliations:** 1 Prosthodontics, CKS Teja Institute of Dental Sciences and Research, Tirupati, IND; 2 Dentistry, Global Clinics, Guntur, IND; 3 Dentistry, Sri Lakshmi Dental Clinic, Hyderabad, IND; 4 Prosthodontics, St. Joseph Dental College, Eluru, IND

**Keywords:** acrylic resins, color, denture base, flexural strength, nanoparticles, polymethyl methacrylate, silver

## Abstract

Background

Polymethyl methacrylate (PMMA) remains the predominant choice for denture base fabrication, primarily due to its superior aesthetic characteristics, excellent biocompatibility with oral tissues, and straightforward processing techniques. However, its relatively low flexural strength and susceptibility to discoloration remain major clinical concerns. Silver nanoparticles (AgNPs), owing to their antimicrobial properties, have been investigated as reinforcing agents, although their influence on the mechanical and optical properties of PMMA remains inconclusive. This study evaluated the effect of different concentrations of AgNPs on the flexural strength and color stability of two heat-polymerized PMMA denture base resins.

Methods

Two commercially available heat-polymerized denture base resins were utilized to prepare 240 standardized PMMA specimens (DPI Heat Cure Denture Base Resin (Dental Products of India Ltd., Mumbai, India) and Pyrax Heat Cure Denture Base Acrylic Resin (Pyrax Polymars, Roorkee, India)). Each resin was divided into four groups: control, 0.5 wt%, 1 wt%, and 2 wt% AgNP incorporation (n = 30/group). Each subgroup comprised 15 specimens for flexural strength testing and 15 for color stability evaluation. Flexural strength was tested using a three-point bending method, and color stability was evaluated with a spectrophotometer (Commission Internationale de l’Éclairage (CIE) L*a*b* color system) before and after 30 days in artificial saliva. Data were analyzed with two-way ANOVA and Tukey’s post hoc test at p < 0.05.

Results

AgNP incorporation significantly affected flexural strength and color stability (p < 0.001). Flexural strength decreased with increasing AgNP concentration in both resin systems, with a greater reduction observed in the Pyrax resin. Color stability was also significantly influenced, with the highest color change observed at 1 wt% AgNP concentration. Significant effects of resin type, nanoparticle concentration, and their interaction were identified for both outcome measures.

Conclusions

AgNP incorporation significantly altered the mechanical and optical properties of heat-polymerized PMMA denture base resins. Optimization of nanoparticle concentration requires careful consideration to maintain acceptable mechanical and aesthetic performance.

## Introduction

Edentulism, characterized by the complete absence of natural teeth, continues to be a major oral health concern, negatively impacting chewing efficiency, speech, facial appearance, nutritional intake, and overall quality of life. It commonly results from dental caries, periodontal disease, trauma, or oral malignancies and is associated with continuous alveolar bone remodeling following tooth loss [1]. Complete dentures continue to be the most widely used and economical treatment option for restoring oral function in completely edentulous patients. The clinical success of complete dentures depends on several factors, including patient-related variables, residual ridge morphology, denture design, fabrication technique, and the material’s physical attributes and mechanical behavior [2].

Heat-cured polymethyl methacrylate (PMMA) has traditionally been regarded as the benchmark material for denture bases, owing to its desirable aesthetic qualities, ease of manipulation, biocompatibility, dimensional reliability, and affordability [3]. Despite these advantages, conventional PMMA exhibits limited mechanical properties, particularly reduced flexural strength and fatigue resistance, making denture fractures one of the most common clinical complications. Repeated masticatory loading, stress concentration, residual processing stresses, and alveolar ridge resorption contribute to crack initiation and propagation, ultimately resulting in denture failure [4,5].

Several strategies have been investigated to enhance the mechanical performance of PMMA, including chemical modification, cross-linking agents, fiber reinforcement, metal wires, and the incorporation of ceramic or metallic fillers such as magnesium oxide, zirconium oxide, and nanoparticles [6]. Among these approaches, silver nanoparticles (AgNPs) have attracted considerable attention because of their reported antimicrobial, physicochemical, and mechanical properties. Previous studies have demonstrated that AgNP incorporation may inhibit *Candida albicans *growth and influence the mechanical properties of PMMA; however, increasing AgNP concentrations may also affect aesthetic properties, particularly color stability [7,8]. Despite these findings, the effects of different AgNP concentrations on the mechanical and optical properties of different commercially available heat-polymerized PMMA formulations remain insufficiently established. Therefore, the objective of the present in vitro study was to comparatively evaluate the effect of incorporating 0.5, 1, and 2 wt% AgNPs on the flexural strength and color stability of two commercially available heat-polymerized PMMA denture base resins (DPI and Pyrax). The study aimed to determine whether the effects of AgNP incorporation varied according to resin type and nanoparticle concentration. It was hypothesized that both resin type and AgNP concentration would significantly influence flexural strength and color stability.

## Materials and methods

Study design

This in vitro experimental investigation was carried out in the Department of Prosthodontics, CKS Teja Institute of Dental Sciences and Research, Tirupati, India, following approval from the Institutional Ethics Committee (ref. no. CKS/PROSTHO/23-24/06). All specimens used in this investigation were laboratory-fabricated PMMA specimens prepared specifically for the experimental procedures in accordance with ISO 20795-1:2013. No specimens were obtained from patients, existing dentures, or biological tissues. Prior to specimen preparation, the required sample size was determined using G*Power software (version 3.1.9.7; Heinrich Heine University, Düsseldorf, Germany) for two-way ANOVA. With an assumed effect size (f) of 0.40, a significance threshold (α) of 0.05, and a statistical power of 80%, the minimum sample size was calculated to be 240 specimens. The specimens were randomly allocated into eight experimental groups (n = 30 per group). Each group was further divided into 15 specimens for flexural strength testing and 15 specimens for color stability evaluation, resulting in a total of 120 specimens for each outcome measure.

Specimen fabrication

Heat-polymerized PMMA denture base resins from two manufacturers were used: DPI Heat Cure Denture Base Resin (Dental Products of India Ltd., Mumbai, India) and Pyrax Heat Cure Denture Base Acrylic Resin (Pyrax Polymars, Roorkee, India). Commercially available AgNPs with a particle size of 10 nm, purity of 99.9%, research grade, and powder form (Nanos Research Lab, Jamshedpur, India) were used as the reinforcing agent.

**Figure 1:**
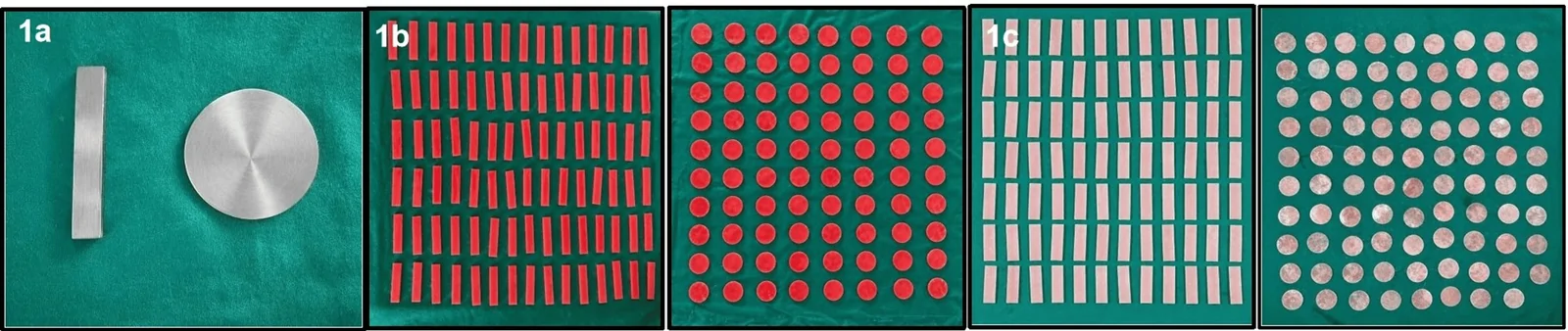
Fabrication and processing of PMMA specimens: (a) stainless-steel molds used for specimen fabrication; (b) wax patterns prepared using the molds; and (c) processed PMMA test specimens after finishing and polishing. PMMA, polymethyl methacrylate

Stainless-steel master molds measuring 65 × 10 × 3 mm were used to prepare rectangular specimens for flexural strength evaluation, while circular molds measuring 50 mm in diameter and 1 mm in thickness were used for color stability assessment (Figure 1a). A thin layer of petroleum jelly was applied to the molds before preparing wax patterns using modeling wax (Figure 1b). The wax patterns were invested in conventional denture flasks with dental plaster. Following complete setting of the investment, dewaxing was carried out in boiling water at approximately 100°C for 15 minutes, after which the mold surfaces were coated with sodium alginate separating medium and allowed to dry (Figure 1c).

Nanoparticle incorporation and processing

AgNPs in powder form (10-g pack; chemical formula Ag; research grade; Nanos Research Lab) were used in the present study. A particle size of 10 nm and purity of 99.9% were reported according to the manufacturer’s specifications. No independent physicochemical characterization of the nanoparticles was performed in the present study. The AgNPs were accurately weighed using a precision electronic analytical balance (Wensar Weighing Scales Ltd., Chennai, India) to obtain concentrations of 0.5 wt%, 1 wt%, and 2 wt%. The nanoparticles were dispersed in the respective PMMA monomer using a REMI 5 MLH digital magnetic stirrer (Remi Elektrotechnik Ltd., Mumbai, India) at 500 rpm for 30 minutes before mixing with the polymer powder. Conventional PMMA without AgNP incorporation served as the control. The polymer and monomer were mixed in a 3:1 polymer-to-monomer ratio by volume, according to the respective manufacturers’ recommendations, until the dough stage was reached. The resin dough was packed into the mold cavities, followed by trial closure to remove excess material and final flask closure. The flasks were bench-cured for 30 minutes and subsequently polymerized using the conventional long curing cycle at 100°C for 1½ hours. Following polymerization, the flasks were allowed to bench-cool for 30 minutes before deflasking to minimize processing-induced stresses.

Finishing and quality assessment

The processed specimens were retrieved from the molds and finished using tungsten carbide acrylic trimming burs. Sequential wet polishing was performed using silicon carbide abrasive papers of 180-, 220-, 400-, and 600-grit under continuous water irrigation, followed by polishing with pumice to obtain smooth and standardized specimen surfaces. Each specimen was visually inspected under adequate illumination by the same investigator, and specimens exhibiting porosity, voids, cracks, dimensional inaccuracies, surface irregularities, or other processing defects were excluded from the study.

Group allocation

The specimens were randomly allocated using a computer-generated randomization sequence into eight experimental groups (n = 30 per group) according to denture base resin type and AgNP concentration. Group A consisted of DPI PMMA specimens: A1 (control), A2 (0.5 wt% AgNP), A3 (1 wt% AgNP), and A4 (2 wt% AgNP). Group B consisted of Pyrax PMMA specimens: B1 (control), B2 (0.5 wt% AgNP), B3 (1 wt% AgNP), and B4 (2 wt% AgNP). Each subgroup comprised 30 specimens, of which 15 specimens were allocated for flexural strength testing and 15 specimens for color stability evaluation. All specimens were coded before testing to minimize observer bias.

Flexural strength evaluation

**Figure 2:**
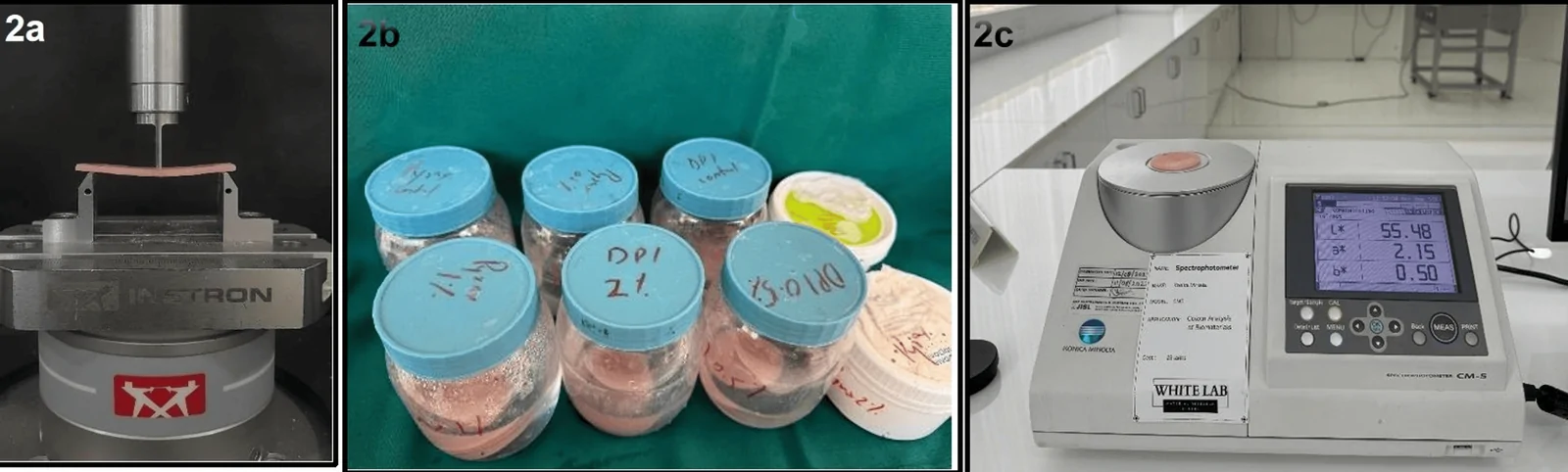
Experimental procedures for evaluation of the study outcomes: (a) PMMA specimens immersed in artificial saliva for color stability evaluation; (b) representative specimens before and after artificial saliva immersion; and (c) three-point bending test of PMMA specimens using a universal testing machine for flexural strength evaluation. PMMA, polymethyl methacrylate

Flexural strength was determined using an Instron Universal Testing Machine (Instron Corp., Norwood, MA, USA) by performing a three-point bending test in accordance with ISO 20795-1:2013. Each specimen was positioned centrally on the supporting jig, and a compressive load was applied until fracture occurred (Figure 2a).

Recorded fracture load values (N) were used to calculate flexural strength (MPa) in accordance with the ISO standard formula:

$\text{Flexural strength (MPa)} = \frac{3WL}{2bd^2},$

where W represents the fracture load (N), L represents the distance between the supports (mm), b represents the specimen width (mm), and d represents the specimen thickness (mm).

Color stability evaluation

Color stability assessment was performed using a CM-5 spectrophotometer (Konica Minolta Sensing, Inc., Osaka, Japan) based on the Commission Internationale de l’Éclairage (CIE) Lab* color system. Prior to each measurement session, the device was calibrated according to the manufacturer’s instructions. Baseline color values (T₀) were obtained by averaging three consecutive readings for each specimen. Subsequently, the specimens were individually immersed in ready-to-use artificial saliva (Nanochemazone, Kurukshetra, India), which has a manufacturer-reported pH of 6.8 and contains sodium carboxymethyl cellulose as a viscosity-modifying agent, and incubated at 37°C for 30 days. The artificial saliva was replaced every evening throughout the immersion period to maintain consistent storage conditions (Figure 2b). Following immersion, the specimens were rinsed with distilled water, gently dried with absorbent paper, and final color measurements (T₁) were recorded under identical conditions. The overall color difference (ΔE) was calculated using the standard CIE Lab* equation (Figure 2c):

$\Delta E = \sqrt{(L_2 - L_1)^2 + (a_2 - a_1)^2 + (b_2 - b_1)^2}$

Statistical analysis

Data analysis was performed with IBM SPSS Statistics for Windows, version 21.0 (released 2012; IBM Corp., Armonk, NY, USA). Results are presented as mean ± standard deviation. The Shapiro-Wilk test was used to assess data normality. Two-way ANOVA was used to examine the effects of resin type and AgNP concentration on flexural strength and color stability. Tukey’s post hoc test was applied for pairwise comparisons, with significance set at p < 0.05.

## Results

Table 1 summarizes the descriptive statistics for flexural strength. In both the DPI and Pyrax PMMA groups, the control specimens exhibited the highest mean flexural strength, with values progressively decreasing as the AgNP concentration increased from 0.5 wt% to 2 wt%. The Pyrax control group showed the highest mean flexural strength (556.92 ± 15.71 MPa), whereas the Pyrax 2 wt% AgNP group recorded the lowest (169.61 ± 21.03 MPa).

**Table 1:** Descriptive statistics of flexural strength (MPa) of heat-polymerized PMMA denture base resins according to resin type and AgNP concentration. Data are presented as mean ± SD (n = 15). AgNP, silver nanoparticle; PMMA, polymethyl methacrylate

Resin	Group	n	Mean ± SD
DPI	Control	15	389.75 ± 5.17
	0.5 wt% AgNP	15	367.46 ± 6.51
	1 wt% AgNP	15	340.38 ± 5.90
	2 wt% AgNP	15	315.59 ± 7.84
Pyrax	Control	15	556.92 ± 15.71
	0.5 wt% AgNP	15	287.11 ± 31.61
	1 wt% AgNP	15	218.30 ± 24.20
	2 wt% AgNP	15	169.61 ± 21.03

Two-way ANOVA demonstrated statistically significant effects of resin type, AgNP concentration, and their interaction on flexural strength (p < 0.001). These findings indicate that the influence of AgNP incorporation differed between the DPI and Pyrax resins (Table 2).

**Table 2:** Two-way ANOVA evaluating the effects of resin type, AgNP concentration, and their interaction on flexural strength. Statistical significance was established at p < 0.05. AgNP, silver nanoparticle; df, degrees of freedom

Source	df	F-value	p-value
Resin type	1	1127.77	<0.001
AgNP concentration	3	1131.45	<0.001
Resin type × AgNP concentration	3	427.01	<0.001

Color stability was influenced by AgNP incorporation in both denture base resins. The maximum color change (ΔE) was observed in the 1 wt% AgNP subgroup for both DPI and Pyrax resins, whereas the control groups exhibited the least color change. Notably, the Pyrax 0.5 wt% AgNP subgroup showed a color change comparable to that of the control group (Table 3).

**Table 3:** Descriptive statistics of color stability (ΔE) of heat-polymerized PMMA denture base resins according to resin type and AgNP concentration. ΔE, color difference; AgNP, silver nanoparticle; PMMA, polymethyl methacrylate

Resin	Group	n	Mean ± SD
DPI	Control	15	0.22 ± 0.01
	0.5 wt% AgNP	15	0.64 ± 0.05
	1 wt% AgNP	15	1.42 ± 0.08
	2 wt% AgNP	15	0.72 ± 0.09
Pyrax	Control	15	0.31 ± 0.02
	0.5 wt% AgNP	15	0.27 ± 0.02
	1 wt% AgNP	15	1.98 ± 0.35
	2 wt% AgNP	15	1.06 ± 0.04

Resin type, AgNP concentration, and the interaction between these variables significantly affected color stability (p < 0.001), indicating that the response to AgNP incorporation differed between the two PMMA resins (Table 4).

**Table 4:** Two-way ANOVA evaluating the effects of resin type, AgNP concentration, and their interaction on color stability (ΔE). ΔE, color difference; AgNP, silver nanoparticle; df, degrees of freedom

Source	df	F-value	p-value
Resin type	1	14.87	<0.001
AgNP concentration	3	120.17	<0.001
Resin type × AgNP concentration	3	59.18	<0.001

The Pyrax control group demonstrated the highest flexural strength, whereas the Pyrax 2 wt% AgNP group exhibited the lowest. For color stability, the lowest ΔE was noted in the DPI control group, while the greatest color change occurred in the Pyrax 1 wt% AgNP group. Overall, increasing AgNP concentration reduced flexural strength and altered color stability, although the magnitude of these effects differed between the two resin systems (Table 5).

**Table 5:** Comparison of flexural strength and color stability among the experimental groups. ΔE, color difference; AgNP, silver nanoparticle

Resin	Group	Flexural strength (mean ± SD)	ΔE (mean ± SD)
DPI	Control	389.75 ± 5.17	0.22 ± 0.01
	0.5 wt% AgNP	367.46 ± 6.51	0.64 ± 0.05
	1 wt% AgNP	340.38 ± 5.90	1.42 ± 0.08
	2 wt% AgNP	315.59 ± 7.84	0.72 ± 0.09
Pyrax	Control	556.92 ± 15.71	0.31 ± 0.02
	0.5 wt% AgNP	287.11 ± 31.61	0.27 ± 0.02
	1 wt% AgNP	218.30 ± 24.20	1.98 ± 0.35
	2 wt% AgNP	169.61 ± 21.03	1.06 ± 0.04

## Discussion

The development of denture base materials has increasingly focused on achieving an optimal balance between mechanical performance, aesthetic stability, and biological functionality. Although modifications using nanoparticles have shown considerable potential for improving the antimicrobial properties of PMMA, their influence on the physical and mechanical characteristics of denture base resins remains inconsistent because it largely depends on the type, concentration, and dispersion of the reinforcing agent within the polymer matrix. Consequently, the incorporation of nanoparticles requires careful evaluation to ensure that any biological advantages are not achieved at the expense of clinically important material properties [9-12]. In view of these considerations, the present study investigated the effect of incorporating AgNPs at concentrations of 0.5 wt%, 1 wt%, and 2 wt% into two commercially available heat-polymerized PMMA denture base resins (DPI and Pyrax).

Flexural strength is considered one of the most important mechanical properties of denture base resins because it reflects the material’s ability to resist deformation and fracture under repeated functional loading. During mastication, dentures are subjected to a combination of tensile, compressive, and shear stresses, making resistance to bending forces essential for long-term clinical performance [13]. In the present study, flexural strength decreased progressively with increasing AgNP concentration in both DPI and Pyrax resins, indicating that nanoparticle incorporation significantly influenced the mechanical behavior of the PMMA matrix. Furthermore, the reduction was more pronounced in the Pyrax resin, suggesting that the effect of AgNP incorporation is material-dependent rather than solely concentration-dependent.

The observed reduction in flexural strength is likely related to the interaction between the nanoparticles and the polymer matrix. The reinforcing effect of nanoparticles depends largely on their homogeneous distribution and effective interfacial adhesion within the resin. Uniformly dispersed nanoparticles can facilitate stress transfer and delay crack propagation, whereas inadequate dispersion results in particle agglomeration that acts as a stress concentration site, promoting crack initiation and reducing the structural integrity of the material. In addition, weak interfacial bonding between AgNPs and the PMMA matrix may impair stress distribution, thereby reducing the material’s resistance to flexural loading [5,14]. Similar observations have been reported in recent systematic reviews evaluating nanoparticle-modified denture base resins, which concluded that the mechanical performance of PMMA is highly dependent on nanoparticle concentration, dispersion, and compatibility with the polymer matrix [9,14].

Another factor that may have contributed to the reduction in flexural strength is the influence of nanoparticles on the polymerization process. The incorporation of metallic nanoparticles can alter polymer chain organization and reduce matrix homogeneity, leading to the formation of microscopic voids or structural irregularities within the resin. These defects may serve as potential sites for crack initiation under cyclic loading and become increasingly significant with higher nanoparticle concentrations. Consequently, although AgNPs offer desirable biological properties, excessive incorporation may adversely affect the continuity of the polymer network and compromise its mechanical performance [15,16].

The findings of the present study are consistent with those reported by Acosta-Torres et al., who demonstrated that the incorporation of AgNPs enhanced the antimicrobial properties of PMMA but did not consistently improve its mechanical properties. They suggested that excessive nanoparticle loading may interfere with the continuity of the polymer matrix, resulting in reduced flexural strength [10]. Similarly, Chladek et al. reported that AgNP-modified acrylic resins exhibited significant antifungal activity; however, they emphasized that increasing nanoparticle concentration could negatively influence the structural properties of the material [6]. A recent systematic review by Galant et al. also concluded that the effect of AgNP incorporation on flexural strength remains concentration-dependent and is strongly influenced by nanoparticle dispersion, particle size, and the interaction between the filler and the resin matrix, highlighting the need for careful optimization before clinical application [14].

The effect of AgNPs on the mechanical properties of PMMA remains controversial, with previous studies reporting improvements, no significant change, or reductions in flexural strength following AgNP incorporation. These variations are likely related to differences in nanoparticle concentration, size, dispersion within the resin matrix, resin composition, and testing protocols [14]. Therefore, direct comparisons among studies should be interpreted with caution, as methodological differences may substantially influence the mechanical behavior of nanoparticle-modified denture base resins.

Besides mechanical performance, maintaining the aesthetic appearance of denture base materials is essential for their long-term clinical success. Color stability directly influences patient satisfaction and the clinical acceptability of complete dentures, as discoloration may necessitate premature replacement despite satisfactory functional performance [17]. In the present study, color stability was evaluated using a spectrophotometer based on the CIE Lab* color system, which provides an objective, reproducible, and sensitive assessment of color differences (ΔE), eliminating the subjectivity associated with visual evaluation. The findings demonstrated that AgNP incorporation significantly influenced the color stability of both heat-polymerized PMMA resins, with the degree of discoloration varying according to nanoparticle concentration and resin type.

The observed color changes may be attributed to the optical properties of AgNPs and their interaction with the PMMA matrix. Metallic nanoparticles can alter light absorption, transmission, and scattering within the resin, while particle agglomeration and water sorption may further modify the refractive index of the polymer, resulting in perceptible color changes [7]. The present findings are consistent with those of Chladek et al. and Acosta-Torres et al., who reported that AgNP incorporation altered the optical characteristics of PMMA despite providing antimicrobial benefits [6,10]. Likewise, a recent systematic review by Galant et al. concluded that the effect of AgNPs on color stability depends on nanoparticle concentration, particle size, dispersion, and compatibility with the polymer matrix [14]. Interestingly, the greatest color change in the present study was observed in the 1 wt% AgNP groups rather than the 2 wt% groups, suggesting that color alteration is influenced not only by nanoparticle concentration but also by their distribution and interaction within the resin matrix.

In contrast, some studies have reported minimal or clinically acceptable color changes following AgNP incorporation, particularly when lower nanoparticle concentrations, surface-modified nanoparticles, or improved dispersion techniques were employed. These variations may be attributed to differences in nanoparticle characteristics, polymerization protocols, aging methods, and resin composition, making direct comparisons difficult [13,18]. The difference in color stability observed between the DPI and Pyrax resins in the present study further supports the influence of resin composition on the optical behavior of nanoparticle-modified PMMA. Therefore, careful optimization of nanoparticle concentration and dispersion is essential to achieve the antimicrobial advantages of AgNPs while maintaining acceptable aesthetic properties for clinical application.

From a practical perspective, the present study provides clinically relevant preliminary information by evaluating both the mechanical and optical behavior of AgNP-modified denture base PMMA. The inclusion of two commercially available heat-polymerized PMMA formulations and three AgNP concentrations allows comparison of whether the effects of AgNP incorporation vary according to resin formulation and nanoparticle concentration. Evaluation under standardized laboratory conditions provides a controlled basis for assessing these changes and may provide useful preliminary information for further investigation of AgNP-modified denture base materials.

This in vitro study has certain limitations. The AgNP particle size and purity were based on the manufacturer’s specifications, and independent physicochemical characterization of the nanoparticles was not performed. Furthermore, the dispersion and potential agglomeration of AgNPs within the PMMA matrix were not independently characterized. The experimental conditions could not fully replicate the complex oral environment, where denture base materials are subjected to fluctuations in temperature, pH, masticatory loading, microbial biofilm formation, and exposure to dietary staining agents. The evaluation was also limited to flexural strength and color stability following 30 days of artificial saliva storage. Other clinically relevant properties, including impact strength, fatigue resistance, wear behavior, surface roughness, antimicrobial efficacy, silver ion release, and long-term biocompatibility, were not investigated. Consequently, mechanistic explanations related to nanoparticle dispersion, agglomeration, and particle-matrix interactions should be interpreted cautiously. Therefore, caution should be exercised when extrapolating these findings directly to clinical practice.

Future research should focus on optimizing the concentration and distribution of AgNPs within PMMA to achieve an appropriate balance between mechanical strength, color stability, and antimicrobial efficacy. Long-term investigations incorporating thermocycling, cyclic fatigue, and extended aging protocols are recommended to better simulate the oral environment. Further studies should also evaluate the biocompatibility, silver ion release, and cytotoxic potential of AgNP-modified denture base resins before clinical application.

## Conclusions

Within the limitations of this in vitro study, AgNP incorporation significantly influenced the flexural strength and color stability of both DPI and Pyrax heat-polymerized PMMA denture base resins. Increasing nanoparticle concentration reduced flexural strength and affected color stability, with the extent of change varying between the two resin systems. These findings suggest that careful consideration of AgNP concentration is necessary to maintain acceptable mechanical and aesthetic performance. Further laboratory and clinical studies are required before routine clinical application.
